# Prognostic Values of Pregnancy-Associated Plasma Protein-A and IGF-Binding Protein-4 Fragments in Patients with Acute Myocardial Infarction

**DOI:** 10.3390/jcm15155760

**Published:** 2026-07-23

**Authors:** Huan Li, Zijian Wang, Yichun Wang, Boyi Bao, Dingkun Wang, Weiping Li

**Affiliations:** 1 Department of Cardiology, Beijing Youan Hospital, Capital Medical University, Beijing 100069, China; perseverqh@163.com; 2 Department of Cardiology, Beijing Aerospace General Hospital, Beijing 100076, China; wangzxj93@126.com; 3 Department of Cardiology, Cardiovascular Center, Beijing Friendship Hospital, Capital Medical University, Beijing 100050, China; yichun_1024@163.com (Y.W.); baoboyi@163.com (B.B.); wangzhengchangzc@163.com (D.W.); 4 Beijing Key Laboratory of Metabolic Disorder Related Cardiovascular Disease, Beijing 100050, China

**Keywords:** pregnancy-associated plasma protein-A, insulin-like growth factor binding protein-4 fragments, acute myocardial infarction, chronic kidney disease, cardiovascular outcome

## Abstract

**Background:** Pregnancy-associated plasma protein-A (PAPP-A)-derived N- and C-terminal fragments of IGF-binding protein-4 (NT- and CT-IGFBP-4) are released from vulnerable atherosclerotic plaques. This study investigated the prognostic value of PAPP-A and IGFBP-4 fragments in patients with acute myocardial infarction (AMI), especially in those combined with chronic kidney disease (CKD). **Methods:** This prospective study measured admission levels of PAPP-A, NT- and CT-IGFBP-4 in 341 consecutive AMI patients. Of these patients, 232 were diagnosed with CKD (AMI and CKD), which consisted predominantly of early-stage cases. The primary outcome was one-year major adverse cardiovascular and cerebrovascular events (MACCE), a composite of cardiovascular death, non-fatal MI, and stroke. **Results:** In the whole AMI cohort and the AMI and CKD subgroup, serum PAPP-A and NT-IGFBP-4 levels in patients with MACCE were both significantly higher than those in patients without MACCE, whereas CT-IGFBP-4 levels were similar. Multivariable Cox regression analysis revealed that increased levels of PAPP-A (HR, 2.48; 95% CI, 1.09–5.63; *p* = 0.030) and NT-IGFBP-4 (HR, 2.48; 95% CI, 1.03–6.02; *p* = 0.044) were associated with MACCE in the whole AMI cohort. The areas under the receiver operating characteristic (ROC) curve for PAPP-A and NT-IGFBP-4 were 0.637 and 0.633, respectively. However, in the AMI and CKD subgroups, only NT-IGFBP-4 was identified as an independent predictor of MACCE (HR, 2.72; 95% CI, 1.16–6.41; *p* = 0.021) and cardiovascular death (HR, 2.63; 95% CI, 1.01–6.84; *p* = 0.048). **Conclusions:** PAPP-A and NT-IGFBP-4 were independently associated with one-year MACCE. NT-IGFBP-4 may be a promising prognostic marker in AMI patients with early-stage CKD.

## 1. Introduction

Acute myocardial infarction (AMI), representing one of the leading causes of cardiovascular morbidity and mortality, often occurs in patients with chronic kidney disease (CKD) [1,2]. The coronary lesions are more diffuse and complex in patients with AMI and CKD than in those with AMI but without CKD [3]. Previous studies have shown that the incidence of adverse events, such as readmission, recurrent MI, revascularization, in-stent restenosis, thrombosis, and all-cause mortality, increases significantly in AMI patients combined with CKD [4,5]. Hence, reliable prognostic biomarkers are critical for developing effective preventive therapies in this population.

Nowadays, pregnancy-associated plasma protein-A (PAPP-A), a metalloproteinase, has been found to be a valuable prognostic predictor in acute coronary syndrome (ACS) [6]. The role of PAPP-A in atherosclerotic plaque destabilization and rupture was confirmed in PAPP-A overexpression and knockout mouse models [7,8]. Some studies have shown that increased PAPP-A levels were associated with a higher all-cause mortality in dialysis patients [9]. However, the prognostic value of PAPP-A in AMI patients with CKD remains unclear. PAPP-A can cleave the insulin-like growth factor binding protein-4 (IGFBP-4) between M135 and K136, resulting in the formation of N- and C-terminal fragments (NT- and CT-IGFBP-4) with molecular masses of 18 and 14 kDa, respectively [10]. The circulating concentrations of these two IGFBP-4 fragments reflect the enzymatically active form of PAPP-A, which is responsible for plaque destabilization, rather than the total PAPP-A level, which includes the inactive PAPP-A/proMBP form. A previous study has demonstrated that truncated or post-translationally modified NT- and CT-IGFBP-4 were absent from the blood of patients with ACS [11]. In addition, PAPP-A assays are technically challenging because PAPP-A is usually present at relatively low concentrations in the blood and is easily influenced by heparin administration. Furthermore, the prognostic value of NT- and CT-IGFBP-4 in AMI patients, especially in those with concomitant CKD, remains far from clear.

To fill these knowledge gaps, we conducted this prospective study to compare the prognostic values of PAPP-A and IGFBP-4 fragments for major adverse cardiovascular and cerebrovascular events (MACCE) in AMI patients, particularly when combined with CKD.

## 2. Materials and Methods

### 2.1. Study Population and Design

**Figure 1:**
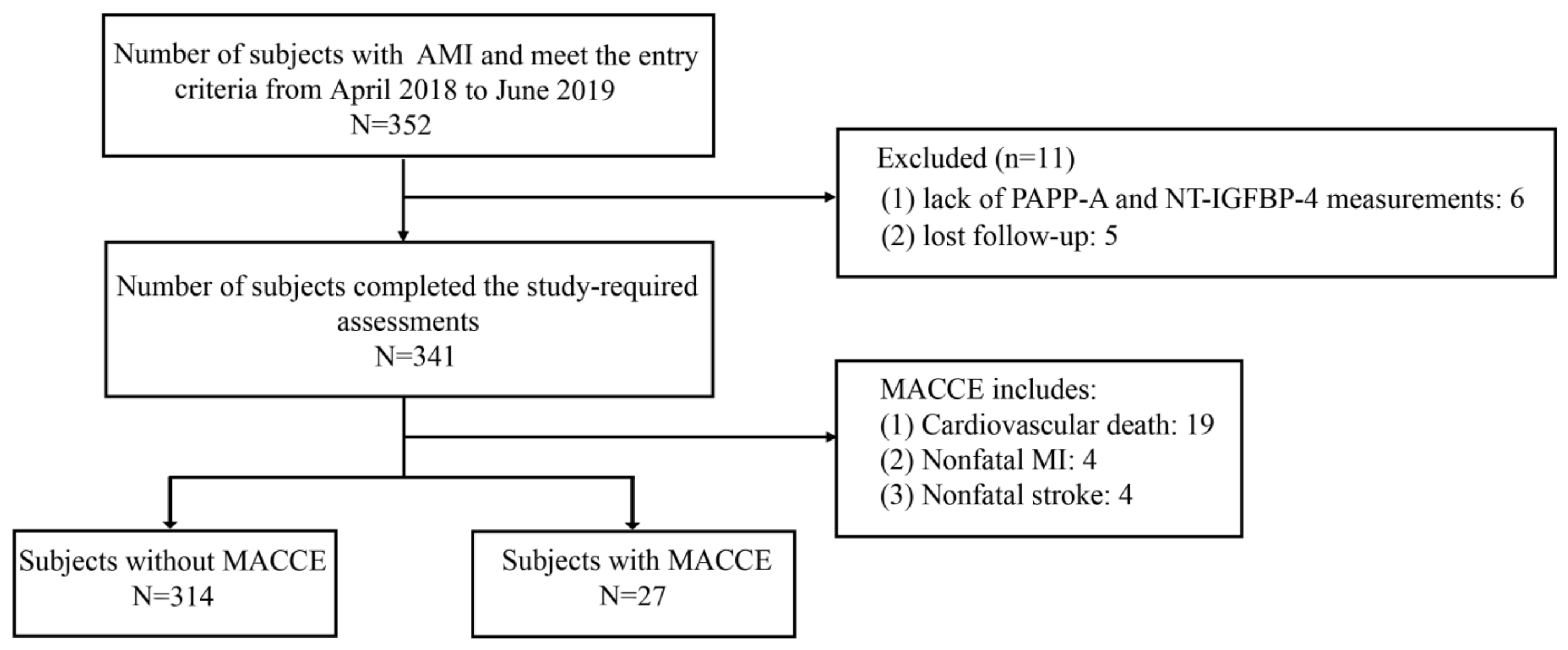
Study subject enrollment and completion.

As shown in Figure 1, a total of 352 consecutive patients with AMI, including ST-elevation myocardial infarction (STEMI) and non-ST-elevation myocardial infarction (NSTEMI), were admitted to Beijing Friendship Hospital between December 2018 and June 2019. The exclusion criteria were as follows: (1) severe liver dysfunction; (2) rheumatic disease or acute infectious disease; (3) current neoplastic disease; (4) an estimated life expectancy of less than 1 year; and (5) pregnancy. Of all the patients, 341 completed the study and were included in the final statistical analysis. Eleven patients were excluded due to missing or incomplete clinical and follow-up data.

AMI was diagnosed based on the fourth universal definition of myocardial infarction (2018) [12]: there is acute myocardial injury with clinical evidence of acute myocardial ischemia and with detection of a rise and/or fall of cardiac troponin (cTn) values with at least one value above the 99th percentile upper reference limit (URL) and at least one of the following: (a) typical symptoms of myocardial ischemia; (b) new ischaemic changes in electrocardiogram; (c) presence of pathological Q waves; (d) imaging evidence of new regional wall motion abnormality or new loss of viable myocardium consistent with an ischaemic etiology; (e) coronary thrombus identified by angiography or autopsy. The diagnosis of STEMI was established when patients develop new ST-segment elevations in two contiguous leads or new bundle branch blocks with ischaemic repolarization patterns. Accordingly, patients without ST-segment elevation are diagnosed with NSTEMI. CKD was diagnosed based on the Kidney Disease: Improving Global Outcomes (KDIGO) 2012 Clinical Practice Guidelines [13] and defined as kidney damage or decreased estimated glomerular filtration rate (eGFR) present for at least 3 months. Markers of kidney damage (one or more) include: (a) albuminuria (albumin excretion rate ≥30 mg/24 h; albumin-to-creatinine ratio ≥30 mg/g or ≥3 mg/mmol); (b) urine sediment abnormalities; (c) abnormalities detected by histology; (d) electrolyte and other abnormalities caused by tubular disorders; (e) history of renal transplantation; (f) structural abnormalities demonstrated by imaging. Decreased eGFR means eGFR < 60 mL/min/1.73 m^2^. The CKD Epidemiology Collaboration (CKD-EPI) equation was used to calculate eGFR in milliliters per minute per 1.73 m^2^ [14]. CKD-EPI = 141 × min [(serum creatinine (SCr)/κ, 1)α × max (SCr/κ, 1)] − 1.209 × 0.993 Age [×1.018 if female] [×1.159 if black], where SCr is serum creatinine (in mg/dL), κ is 0.7 for females and 0.9 for males, α is 0.329 for females and 0.411 for males, min is the minimum of SCr/κ or 1, and max is the maximum of SCr/κ or 1.14. Patient demographic data, medical and medication history, and laboratory results were collected and verified through electronic medical records.

The study was approved by the Beijing Friendship Hospital Institutional Review Board and conducted in compliance with the Declaration of Helsinki. Written informed consent was obtained from all the study participants.

### 2.2. Laboratory Analysis

Venous blood samples were collected at admission and stored at −80 °C. All routine laboratory tests were performed using standardized methods at the Beijing Friendship Hospital Laboratory. Serum PAPP-A, NT-IGFBP-4, and CT-IGFBP-4 levels were measured before heparin administration. An ultrasensitive enzyme-linked immunosorbent assay (ELISA) kit for PAPP-A was obtained from DRG Instruments GmbH (Marburg, Germany) [15]. The assay utilized a mouse anti-human PAPP-A monoclonal capture antibody. The standard curve ranged from 0.02 to 500 ng/mL (R^2^ = 0.994) with intra- and inter-assay coefficients of variation (CVs) <5% and <8.5%, respectively. Immunoassay kits for CT-IGFBP-4 and NT-IGFBP-4 were performed using commercially available ELISA kits (BLUE GENE, Shanghai, China). CT-IGFBP-4 and NT-IGFBP-4 were measured via ELISA with goat anti-human C-terminal IGFBP-4 polyclonal antibody and goat anti-human N-terminal IGFBP-4 polyclonal antibody, with intra- and inter-assay CVs <5% and <7% for NT-IGFBP-4, and <5% and <8% for CT-IGFBP-4, respectively. The assay demonstrated a limit of detection (LOD) of 1.0 pg/mL and linearity from 1 to 5000 pg/mL (R^2^ = 0.999). QC samples were included in each run to ensure precision. Samples underwent one freeze–thaw cycle prior to analysis, and the assays were conducted between December 2018 and July 2019 using an EL × 800 microplate reader (BioTek Instruments, Inc., Winooski, VT, USA). QC samples were included in each run to ensure precision. Laboratory personnel performing the assays were blinded to all clinical outcomes and patient information.

### 2.3. Echocardiograms

Transthoracic echocardiography was performed within the first 24 h of admission. All images were analyzed on the EchoPAC workstation (version 112; GE Healthcare, Chicago, IL, USA), and images were acquired on a Philips iE Elite system (Philips Healthcare, Andover, MA, USA). Left ventricular end-diastolic and end-systolic volumes were measured using Simpson’s method in the apical two- and four- chamber views, as recommended by the American Society of Echocardiography [16]. Left ventricular ejection fraction (LVEF) was calculated according to the formula: [(end-diastolic volume − end-systolic volume)/end-diastolic volume] × 100%.

### 2.4. Patient Follow-Up and End Points

The follow-up visits were conducted by telephone for months 1, 3, 6, and 12 after discharge. The study primary endpoint was the composite of MACCE, including cardiovascular (CV) death, non-fatal MI, or non-fatal stroke. For those with recurrent events, the first event and its time of occurrence were recorded. CV death was defined as sudden death, fatal myocardial infarction, fatal stroke, and other cardiovascular deaths. Stroke, including ischemic and hemorrhagic stroke, was defined as cerebral dysfunction caused by cerebral vascular obstruction or sudden rupture, and was diagnosed based on symptoms and signs of neurological dysfunction and/or brain imaging evidence. The median follow-up duration for the entire AMI cohort was 374 days (IQR: 369–382). Telephone-reported events were confirmed by at least two independent follow-up personnel and verified through medical record review and death certificates when available, with adjudicators blinded to biomarker concentrations.

### 2.5. Statistical Analysis

Depending on the distribution of the data, continuous variables are expressed as mean ± standard deviation (SD) or median with interquartile range (IQR). Categorical variables are presented as numbers and percentages. Comparisons of clinical characteristics and laboratory test results between the patients with and without MACCE were performed using Student’s *t*-test or Wilcoxon rank sum test for continuous variables and the Chi-square test or Fisher’s exact test for categorical variables. Receiver operating characteristic (ROC) curves were generated to demonstrate the predictive value of PAPP-A, NT-IGFBP-4, CT-IGFBP-4, and other variables. Cutoff values derived from the ROC curves were defined as the values that provided the best combination of specificity and sensitivity. Cumulative event rates were estimated by Kaplan–Meier curves and were compared using the log-rank test between different groups stratified by the cutoff values of PAPP-A, NT-IGFBP-4, and CT-IGFBP-4. We used the multivariate Cox proportional hazards model to estimate the hazard ratios (HRs) of composite MACCE and CV death in relation to PAPP-A and NT-IGFBP-4 levels. Patients with PAPP-A or NT-IGFBP-4 levels lower than the cutoff values were considered as the reference groups in these models. The following variables were included in the regression models: age, sex, history of stroke, the proportion of STEMI, eGFR, and LVEF. The primary analyses were performed using SPSS 23.0 (IBM Inc, Armonk, NY, USA), MedCalc 14.1 (Mariakerke, Belgium), and GraphPad Prism v8.0d (GraphPad Software, San Diego, CA, USA). As sensitivity analyses, biomarkers were also evaluated as continuous variables (log-transformed, per SD increase); the lack of significant linear associations supported the use of Youden-derived dichotomized thresholds as the primary framework. To mitigate the risk of overfitting due to the limited number of events, internal validation via 1000 bootstrap iterations was performed using R software (version 4.3.1; R Foundation for Statistical Computing, Vienna, Austria), and reported optimism-corrected C-index, Brier score, and Net Reclassification Improvement (NRI). All tests were two-tailed, and *p* < 0.05 was considered statistically significant.

## 3. Results

### 3.1. Clinical and Laboratory Characteristics

Table 1 shows the baseline clinical characteristics and laboratory results. In the whole AMI cohort, MACCE occurred in 27 (7.9%) patients (19 CV deaths, 4 non-fatal MI, and 4 non-fatal strokes) during one-year follow-up. Compared with patients without MACCE, those with MACCE were older (*p* = 0.002), had a higher proportion of previous stroke (*p* = 0.001), and a lower proportion of STEMI at admission (*p* = 0.016), and significantly lower eGFR (81.7 mL/min/1.73 m^2^ vs. 98.0 mL/min/1.73 m^2^, *p* < 0.001) and LVEF (54% vs. 60%, *p* = 0.022). There was no significant difference in previous medication and in-hospital treatment between the two groups.

In the subgroup of 232 patients with AMI with CKD (AMI and CKD), patients with eGFR < 60 mL/min/1.73 m^2^ (CKD Stage 3+): *n* = 43 (19%), patients with eGFR ≥ 60 but with documented albuminuria (CKD Stage 1–2): *n* = 189 (81%). In the subgroup, MACCE occurred in 23 (9.9%) patients (18 CV deaths, 3 non-fatal MI, and 2 non-fatal strokes). Patients with MACCE were older and more frequently had a history of stroke (*p* = 0.003) but were less often diagnosed with STEMI at admission (*p* = 0.001) than those without MACCE; they also had significantly lower eGFR (64.8 mL/min/1.73 m^2^ vs. 88.8 mL/min/1.73 m^2^). The previous medication and in-hospital treatment were similar in the two groups (Table 1).

**Figure 2:**
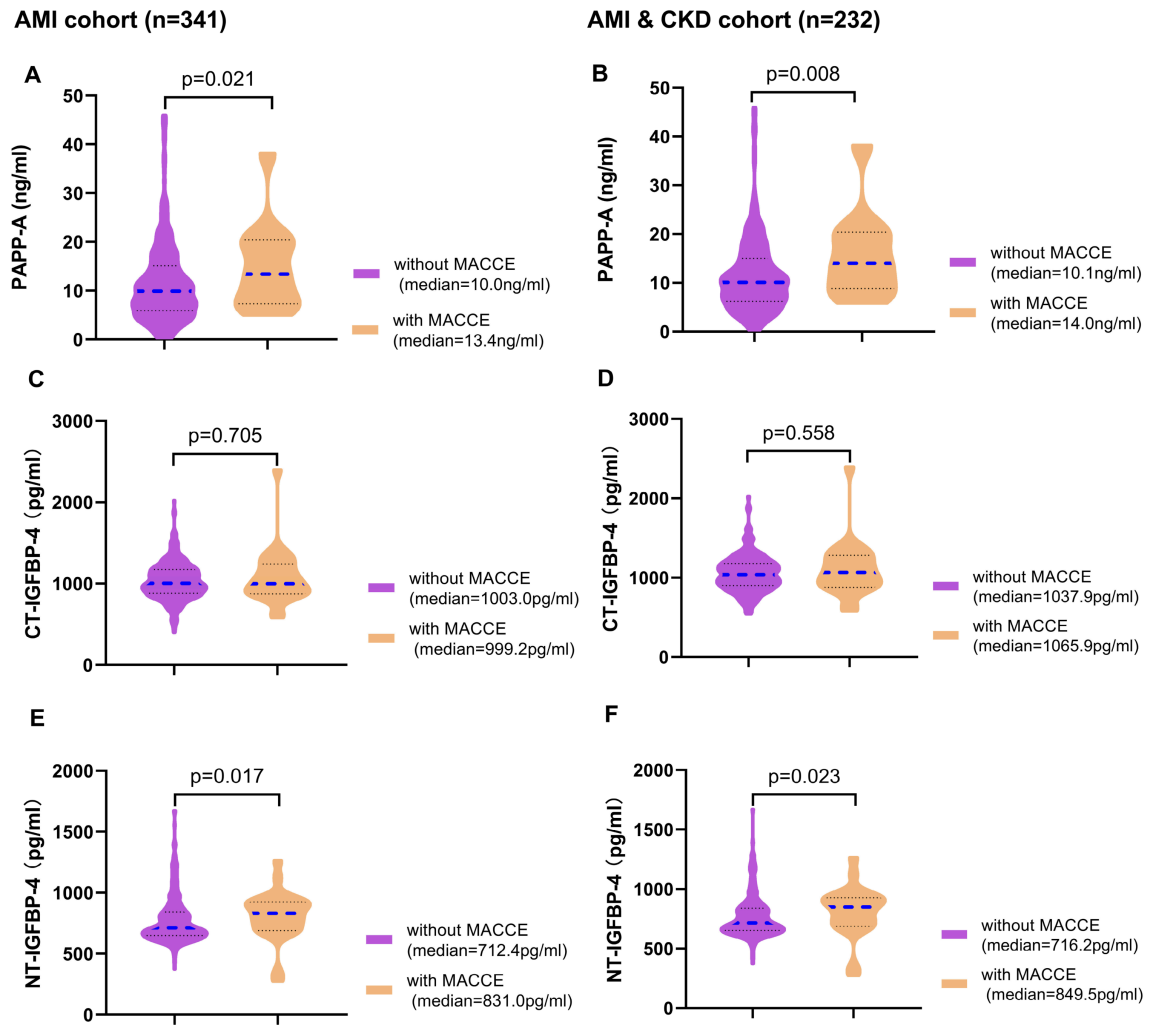
Serum levels of PAPP-A, CT-IGFBP-4 and NT-IGFBP-4 in the studied cohort. PAPP-A level (**A**,**B**), CT-IGFBP-4 level (**C**,**D**), NT-IGFBP-4 level (**E**,**F**) in the whole AMI cohort (*n* = 341) and in the AMI and CKD cohort (*n* = 232), respectively. Abbreviations: PAPP-A, pregnancy-associated plasma protein-A; NT-IGFBP-4, N-terminal fragments of human insulin-like growth factor binding protein-4; CT-IGFBP-4, C-terminal fragments of human insulin-like growth factor binding protein-4.

The violin plots (Figure 2) illustrated PAPP-A, CT-IGFBP-4 and NT-IGFBP-4 levels in the studied cohorts. In the whole AMI cohort, compared with the patients without MACCE, the patients with MACCE had significantly higher PAPP-A levels (13.4 [7.8, 20.6] ng/mL vs. 10.0 [5.0, 15.1] ng/mL, *p* = 0.021, Figure 2A) and higher NT-IGFBP-4 levels (831.0 [689.0, 923.5] pg/mL vs. 712.4 [649.2, 843.9] pg/mL, *p* = 0.017, Figure 2E) whereas similar CT-IGFBP-4 levels (999.2 [873.3, 1240.6] pg/mL vs. 1003.0 [880.0, 1173.7] pg/mL, *p* = 0.705, Figure 2C). Likewise, in the AMI and CKD cohort, median levels of PAPP-A (14.0 [8.8–20.9] ng/mL vs. 10.1 [6.2, 15.2] ng/mL; *p* = 0.008, Figure 2B) and NT-IGFBP-4 (849.5 [687.4, 928.1] pg/mL vs. 716.2 [651.7, 842.5] pg/mL; *p* = 0.023, Figure 2F) were also significantly higher in patients with MACCE than in those without MACCE, and the levels of CT-IGFBP-4 were similar between the 2 groups (Figure 2D).

### 3.2. Predictive Values of PAPP-A and NT-IGFBP-4 Levels on MACCE

**Figure 3:**
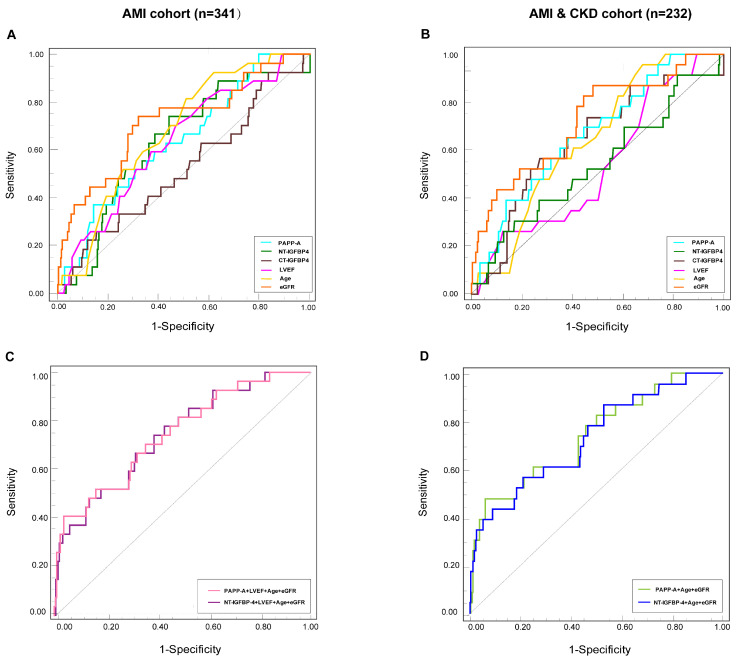
Predictors of MACCE one year post AMI. Receiver operating characteristic (ROC) curves for PAPP-A, NT-IGFBP-4, CT-IGFBP-4, LVEF, Age, eGFR in the whole AMI cohort (**A**,**C**), and in the AMI and CKD cohort (**B**,**D**). Abbreviations: see Table 1, Figure 1.

AUC for PAPP-A, NT-IGFBP-4, CT-IGFBP-4, LVEF, age, eGFR, and the combination of these predictors (from a multivariate logistic regression including all predictors) was presented in Figure 3 and Table 2. In the whole AMI cohort (Figure 3A,C, Table 2), PAPP-A, NT-IGFBP-4, LVEF, age, and eGFR were significant predictors, with AUC of 0.637 (95% CI 0.534–0.740), 0.633 (95% CI 0.531–0.736), 0.633 (95% CI 0.529–0.737), 0.678 (95% CI 0.590–0.766), 0.719 (95% CI 0.610–0.828), respectively. Combining PAPP-A or NT-IGFBP-4 with other factors improved the AUCs to 0.742 (95% CI 0.641–0.841) and 0.738 (95% CI 0.641–0.841), respectively; however, these improvements were not statistically significant compared with the AUCs of PAPP-A or NT-IGFBP-4 alone.

Similarly, in the AMI and CKD cohort (Figure 3B,D, Table 3), PAPP-A, NT-IGFBP-4, age, and eGFR remained significant factors, with AUC of 0.667 (95% CI 0.558–0.777), 0.638 (95% CI 0.517–0.759), 0.641 (95% CI 0.543–0.738), 0.721 (95% CI 0.607–0.834), respectively, whereas LVEF was not a significant predictor. Combining PAPP-A or NT-IGFBP-4 with other factors improved the AUCs to 0.729 (95% CI 0.618–0.840) and 0.726 (95% CI 0.616–0.832), respectively, but these improvements were not statistically significant compared with the AUCs of PAPP-A or NT-IGFBP-4 alone.

### 3.3. Independent Association of PAPP-A and NT-IGFBP-4 Levels with MACCE in Different Cohorts

**Figure 4:**
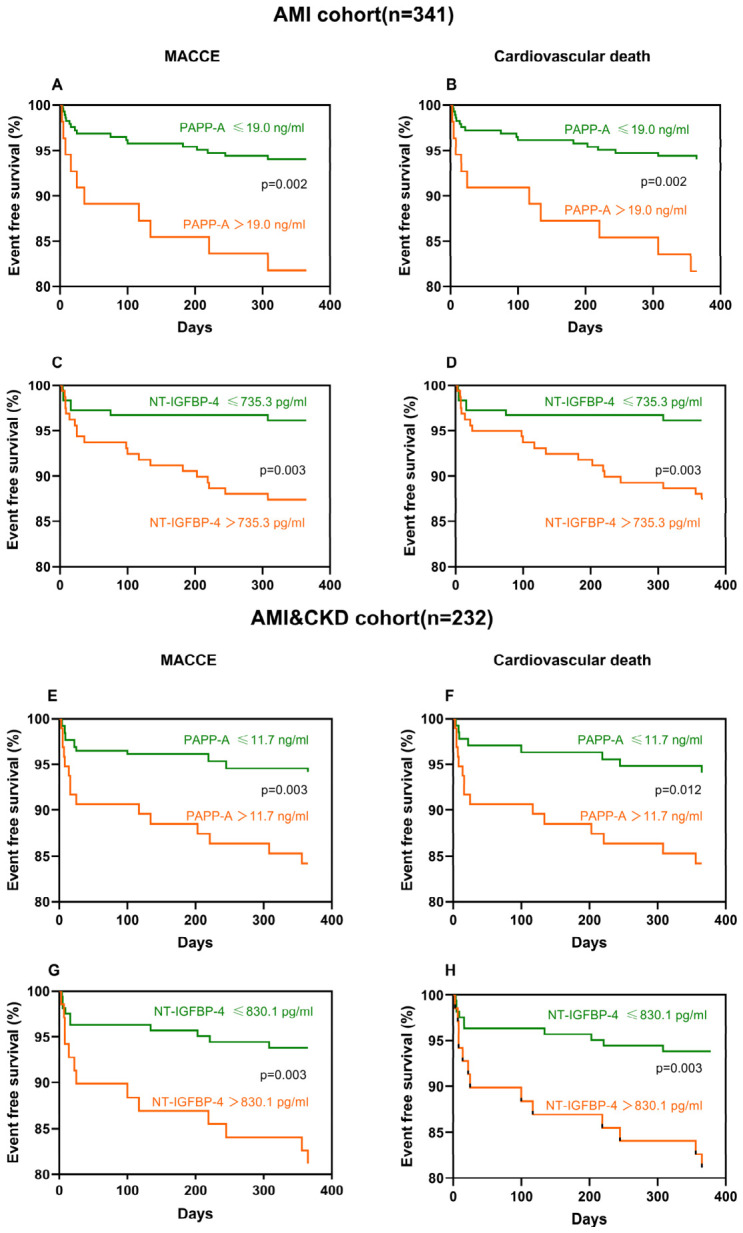
Kaplan–Meier curves for the one-year MACCE and cardiovascular death stratified by the cutoff values of PAPP-A and NT-IGFBP-4 in the whole AMI cohort (**A**–**D**) and AMI and CKD cohort (**E**–**H**). Abbreviations see Table 1.

According to the ROC curve analysis, the PAPP-A and NT-IGFBP-4 cutoff values that best discriminated patients with and without MACCE were as follows: PAPP-A >19.0 ng/mL and NT-IGFBP-4 >735.3 pg/mL in the whole AMI cohort, PAPP-A >11.7 ng/mL and NT-IGFBP-4 >830.1 pg/mL in the AMI and CKD cohort. Figure 4 displays Kaplan–Meier curves for the one-year MACCE and CV death using the pre-defined cutoff values of PAPP-A and NT-IGFBP-4 in the two cohorts. The MACCE-free survival was not significantly different between patients with CT-IGFBP-4 levels above and below the cutoff value.

Table 4 presents HRs for the composite MACCE and CV death calculated using the pre-defined cutoff values of PAPP-A and NT-IGFBP-4 levels. In the unadjusted model, PAPP-A and NT-IGFBP-4 levels were predictive of the incidence of MACCE and CV death in the whole AMI cohort and the AMI and CKD cohort. In the whole AMI cohort, after adjustment for age, sex, the diagnosis of STEMI, prior stroke, eGFR, and LVEF, the patients with PAPP-A levels >19.0 ng/mL had a significantly higher risk of MACCE (HR 2.48, 95% CI 1.09–5.63, *p* = 0.030) and CV death (HR 2.87, 95% CI 1.11–7.45, *p* = 0.030), and the patients with NT-IGFBP-4 levels >735.3 pg/mL had a significantly higher risk of MACCE (HR 2.48, 95% CI 1.03–6.02, *p* = 0.044). However, in the AMI and CKD cohort, PAPP-A levels no longer independently predicted the occurrence of MACCE and CV death after adjusting for the potential confounding factors (age, sex, the diagnosis of STEMI, prior stroke, and eGFR). Notably, NT-IGFBP-4 levels >830.1 pg/mL remained an independent predictor for MACCE (HR 2.72, 95% CI 1.16–6.41, *p* = 0.021) and CV death (HR 2.63, 95% CI 1.01–6.84, *p* = 0.048). To assess model stability, we performed bootstrap internal validation (Supplementary Table S1). For PAPP-A, the optimism-corrected C-index was 0.622 (Brier score: 0.030) in the AMI cohort and 0.110 (Brier score: 0.023) in the AMI and CKD cohort; for NT-IGFBP-4, the C-index was 0.681 (Brier score: 0.029) in the AMI cohort and 0.654 (Brier score: 0.036) in the AMI and CKD cohort. This internal validation thereby supports the reliability of our primary findings.

## 4. Discussion

In this prospective observational study, we evaluated the prognostic value of PAPP-A and its IGFBP-4 cleavage fragments in AMI patients, with a particular focus on those with concomitant CKD. Our findings suggest that NT-IGFBP-4 may offer prognostic information in this population, particularly in early-stage CKD, despite modest standalone discrimination.

PAPP-A, the key component of the PAPP-A/IGFBP-4/IGF-1 axis, is highly expressed in atherosclerotic plaques, and its role in the development of vulnerable atherosclerotic plaque has been demonstrated in PAPP-A transgenic and gene knockout animal models [7]. PAPP-A shows proteolytic activity primarily towards IGFBP-2, IGFBP-4, and IGFBP-5, and closely interacts with IGFBP-4, which is a key regulator of IGF bioactivity. Previous studies have shown that PAPP-A might be a candidate biomarker for the diagnosis and prognosis evaluation in patients with ACS [17,18]. A recent study suggests that PAPP-A may be a potential target for improving the morbidity and mortality of CVD [19]. However, AMI arises from heterogeneous substrates, including plaque rupture and erosion. Notably, plaque erosion is characterized by less inflammation than rupture [20], which may partly explain the modest predictive performance of PAPP-A and its fragments observed in our study.

Patients with CKD have a poorer prognosis and elevated cardiovascular risk. Previous studies have demonstrated that PAPP-A has prognostic potential in long-term hemodialysis patients [21], renal transplant recipients [22], and patients with diabetic nephropathy [23]. It has been suggested as a valuable biomarker associated with cardiovascular dysfunction, malnutrition and inflammatory state in hemodialysis patients [24]. However, the precise role of PAPP-A in AMI patients with early-stage CKD remains unclear. In the present study, after adjustment for conventional risk factors, PAPP-A was no longer an independent predictor of MACCE in the AMI and CKD subgroup—a discrepancy that may be explained by the different population profile, as 81% of patients in our cohort had early-stage CKD and only 5% were on dialysis. Notably, NT-IGFBP-4, the downstream cleavage product of PAPP-A activity, emerged as a potential prognostic marker in this population.

IGFBP-4 fragments, the cleavage products of PAPP-A activity, have been considered as a novel marker of cardiovascular diseases. Compared with measuring PAPP-A itself, we proposed that quantifying the downstream products of its enzymatic activity—IGFBP-4 fragments—may provide a more direct assessment of PAPP-A bioavailability and thus offer higher clinical value. First, heparin administration artificially increases circulating PAPP-A levels by competing for cell-surface binding sites and releasing tissue-bound PAPP-A into the circulation [10]. In contrast, endogenous NT-IGFBP-4 and CT-IGFBP-4 are stable in storage for up to 20 years and do not undergo any modification, truncation or complex formation in the blood [11,25] and are also not affected by heparin or PCI treatment [26]. Second, most commercial PAPP-A assays cannot distinguish between active and inactive PAPP-A (the PAPP-A/proMBP complex), whereas IGFBP-4 fragments reflect the total concentration of the active PAPP-A, regardless of its localization-both in the circulation and within atherosclerotic plaques. Third, circulating IGFBP-4 fragment levels far exceed those of PAPP-A, facilitating more accurate and reliable measurement with reduced sample volume [25].

So far, studies of IGFBP-4 fragments as cardiovascular risk markers remain limited and have yielded conflicting results. In patients with stable cardiovascular diseases, neither NT-IGFBP-4 nor CT-IGFBP-4 was found to be a predictor of long-term all-cause and cardiovascular mortality [27]. By contrast, in higher-risk populations, positive associations have been reported: both fragments were predictive of 6-month MACE in patients with suspected myocardial ischemia [28]; NT- and CT-IGFBP-4 were associated with all-cause death, cardiovascular death, and MACE in STEMI patients [29]; and intact IGFBP-4 predicted adverse outcomes during long-term follow-up in STEMI patients treated with primary PCI [30]. Additionally, elevated CT-IGFBP-4 was independently associated with one-year all-cause mortality in acute heart failure [31], and both fragments were associated with increased mortality in type 1 diabetes patients with or without diabetic nephropathy over 12 years of follow-up [25]. These discrepancies may reflect differences in study populations, disease acuity, and follow-up duration. Nevertheless, to date, the prognostic role of IGFBP-4 fragments in AMI patients combined with CKD remains unclear.

In the present study, we measured PAPP-A and IGFBP-4 fragment levels before heparin administration to evaluate their prognostic value in AMI patients. In the whole AMI cohort, both PAPP-A and NT-IGFBP-4 were significantly associated with one-year MACCE after multivariable adjustment. However, in the AMI and CKD subgroup, PAPP-A lost its predictive value, whereas NT-IGFBP-4 remained independently associated with MACCE and CV death. This finding was supported by bootstrap validation, which confirmed moderate but stable performance for NT-IGFBP-4 (C-index: 0.654), while PAPP-A showed no predictive utility in this subgroup (C-index: 0.110). Recently, Dang et al. investigated inflammatory markers in CKD patients with AMI and reported that the neutrophil-to-lymphocyte ratio (NLR) was independently associated with in-hospital MACCE (OR 10.764, AUC 0.748) [32]. While their study focused on acute inflammatory response, our study evaluated markers of plaque vulnerability (PAPP-A and IGFBP-4 fragments) and demonstrated their association with 1-year MACCE. Together, these complementary findings support a multi-biomarker approach combining inflammatory and plaque-related markers for risk stratification in this population.

The difference in the predictive value of NT-IGFBP-4 and CT-IGFBP-4 fragments may be explained by differences in their metabolism. Although both the N-terminal and C-terminal domains of IGFBP-4 contain IGF-binding sites, the N-terminal domain appears to be the main IGF-binding site and thus has a higher affinity for IGFs, which facilitates more efficient interaction between IGFs and cellular IGF receptors [33,34]. Thus, NT-IGFBP-4 levels, which reflect the degree of IGFBP-4 proteolysis, may provide a better measurement of the PAPP-A activity in vivo than PAPP-A levels per se. Furthermore, differential metabolic stability is likely a key factor. Although both fragments are small molecules (<30 kDa) and theoretically susceptible to renal clearance, the CT-IGFBP-4 fragment may be cleared more rapidly or undergo further degradation, resulting in greater variability in its plasma levels. In contrast, the NT-IGFBP-4 fragment may have a longer half-life, possibly due to post-translational modifications such as glycosylation, which has been demonstrated in ACS patients and may protect the fragment from proteolysis [35]. Thus, NT-IGFBP-4 may serve as a more stable and reliable indicator of PAPP-A activity. These findings support that measurement of NT-IGFBP-4 may assist in the risk stratification of AMI patients, particularly in those with CKD.

Several limitations of our study should be acknowledged. First, as a single-center study with a moderate sample size and a limited number of endpoint events, the statistical power is constrained. This necessitates cautious interpretation of the findings, particularly in subgroup analyses, despite bootstrap validation supporting the stability of our primary results. These findings warrant prospective external validation in larger, multi-center cohorts. Second, due to the limited number of patients with advanced CKD (stage 3+) and the consequent low event rate in this subgroup, our findings regarding the prognostic utility of NT-IGFBP-4 are primarily indicative of its value in patients with earlier stages of CKD. Future studies with larger cohorts are needed to explore its role in advanced CKD. Third, the study population was not completely homogeneous, as both NSTEMI and STEMI patients were enrolled. However, we adjusted for STEMI diagnosis in the multivariable Cox regression models for MACCE and CV death in both cohorts. Moreover, given the increasing prevalence of NSTEMI and the similarly high risk of recurrent cardiovascular events in these patients, we consider it important to include this population in prognostic analyses. Fourth, serum biomarker levels were measured only once at baseline rather than at multiple time points. Finally, the follow-up duration was limited to one year, and longer follow-up is required to confirm the long-term predictive value of PAPP-A and IGFBP-4 fragments.

## 5. Conclusions

PAPP-A and NT-IGFBP-4 were independently associated with the one-year risk of MACCE in patients with AMI. Notably, NT-IGFBP-4 also showed a potential association with outcomes in AMI patients with early-stage CKD, whereas CT-IGFBP-4 levels were not associated with MACCE. Further studies with larger cohorts are warranted to confirm these findings and to validate the prognostic value of NT-IGFBP-4 in this population.

## Figures and Tables

**Table 1:** Baseline clinical characteristics and laboratory assessment of the studied cohorts relative to the presence of MACCE.

Variable	Patients with AMI (*n* = 341)			Patients with AMI & CKD (*n* = 232)		
	Without MAACE (*n* = 314)	With MACCE (*n* = 27)	*p* Value	Without MACCE (*n* = 209)	With MACCE (*n* = 23)	*p* Value
Age, years	62 (56, 70)	70 (62, 77)	0.002	64 (56, 75)	71 (62, 78)	0.027
Male, *n* (%)	240 (76)	21 (78)	0.874	154 (74)	18 (78)	0.634
Physical sign						
BMI, kg/m^2^	25.4 (23.4, 27.7)	26.0 (24.2, 26.6)	0.895	25.3 (23.3, 27.8)	26.0 (24.5, 27.6)	0.449
SBP, mmHg	125 (112, 141)	121 (112, 132)	0.735	125 (112, 142)	121 (112, 145)	0.777
DBP, mmHg	72 (62, 80)	74 (63, 84)	0.574	72 (62, 80)	74 (63, 84)	0.670
HR, bpm	75 (65, 86)	77 (72, 84)	0.291	74 (65, 85)	77 (70, 89)	0.298
Medical history						
Hypertension	199 (63)	21 (78)	0.133	142 (68)	20 (87)	0.059
Diabetes mellitus	123 (39)	14 (52)	0.197	90 (43)	13 (57)	0.218
Hyperlipidemia	151 (48)	14 (52)	0.707	107 (51)	11 (48)	0.759
Previous CAD	86 (27)	9 (33)	0.508	61 (29)	8 (35)	0.577
Previous MI	44 (14)	4 (15)	1.000	30 (14)	4 (17)	0.755
Previous stroke	43 (14)	10 (37)	0.001	32 (15)	10 (44)	0.003
Smoking	197 (63)	15 (56)	0.460	123 (59)	13 (57)	0.830
Previous medical therapy						
Antiplatelet agent	74 (24)	8 (30)	0.479	45 (22)	6 (26)	0.617
ACEI/ARBs	89 (28)	10 (37)	0.340	68 (33)	9 (39)	0.524
Beta-blocker	38 (12)	4 (15)	0.758	32 (15)	4 (17)	0.764
Statins	27 (9)	5 (19)	0.157	18 (9)	4 (17)	0.248
Diagnosis at admission						
STEMI	169 (54)	8 (30)	0.016	119 (57)	5 (22)	0.001
Main epicardial coronary arteries >75% stenosis						
1	264 (84)	21 (78)	0.713	179 (86)	17 (74)	1.000
2	124 (39)	10 (37)	0.744	84 (40)	10 (43)	0.308
3	44 (14)	5 (19)	0.355	33 (16)	5 (22)	0.329
Left main ≥50% stenosis	35 (11)	2 (7)	1.000	20 (10)	2 (9)	1.000
In-hospital treatment						
PCI/CABG	265 (84)	19 (70)	0.101	165 (79)	16 (70)	0.302
Antiplatelet agent	298 (95)	25 (93)	0.644	200 (96)	21 (91)	0.299
ACEI/ARBs	214 (68)	15 (56)	0.202	147 (70)	13 (57)	0.234
Beta-blocker	230 (75)	20 (74)	1.000	162 (78)	17 (74)	0.696
Statins	275 (88)	21 (78)	0.146	181 (87)	18 (78)	0.340
Laboratory assessment						
HbA1c, (%)	6.1 (5.7, 7.3)	6.5 (5.8, 7.5)	0.293	6.3 (5.8, 7.8)	6.5 (5.9, 7.5)	0.548
FPG, mmol/L	6.0 (5.2, 8.2)	6.4 (5.6, 8.8)	0.200	6.3 (5.3, 8.5)	6.4 (5.7, 8.8)	0.401
eGFR, mL/min/1.73 m^2^	98.0 (81.0, 108.5)	81.7 (40.7, 94.1)	<0.001	88.8 (68.6, 105.1)	64.8 (18.6, 84.3)	0.001
hsCRP, mg/L	10.7 (2.7, 28.2)	22.8 (2.8, 42.3)	0.125	12.4 (2.8, 31.1)	27.6 (6.6, 44.8)	0.065
TC, mmol/L	4.32 (3.75, 5.02)	3.98 (3.33, 4.81)	0.129	4.20 (3.70, 5.51)	3.90 (3.30, 4.63)	0.116
TG, mmol/L	1.38 (0.98, 2.00)	1.25 (0.91, 1.71)	0.308	1.42 (0.99, 2.01)	1.25 (0.91, 1.71)	0.367
HDL-C, mmol/L	1.03 (0.90, 1.20)	0.98 (0.82, 1.18)	0.328	1.02 (0.89, 1.19)	0.98 (0.80, 1.17)	0.327
LDL-C, mmol/L	2.45 (2.01, 3.02)	2.39 (1.78, 2.83)	0.343	2.42 (1.98, 2.99)	2.30 (1.67, 2.83)	0.288
CK-MB peak, u/L	28.15 (9.4, 87.2)	29.2 (5.4, 94.5)	0.765	32.9 (9.1, 89.4)	29.2 (6.7, 94.5)	0.909
cTnI peak, ng/mL	14.63 (2.2, 50.0)	13.8 (1.8, 40.7)	0.713	18.3 (3.15, 50.0)	13.8 (2.3, 44.3)	0.686
cTnT peak, ng/mL	1.6 (0.4, 3.8)	1.5 (0.4, 3.9)	0.988	2.0 (0.4, 4.5)	1.7 (0.4, 3.9)	0.954
LVEF, (%)	60 (52, 64)	54 (46, 61)	0.022	58 (49, 63)	54 (44, 61)	0.071

Values are median (interquartile range) or *n* (%). Wilcoxon Rank-sum test was used for comparisons of continuous variables. Abbreviations: MACCE, major adverse cardiovascular and cerebrovascular events; BMI, body mass index; SBP, systolic blood pressure; DBP, diastolic blood pressure; HR, heart rate; CAD, coronary artery disease; AMI acute myocardial infarction; STEMI, ST segment elevation myocardial infarction; PCI, percutaneous coronary intervention; CABG, coronary artery bypass graft; ACEI, angiotensin converting enzyme inhibitor; ARB, angiotensin-receptor blocker; HbA1c, hemoglobin A1c; FPG, fasting plasma glucose; eGFR, estimated glomerular filtration rate; hsCRP, high-sensitivity C-reactive protein; TC, total Cholesterol; TG, triglyceride; HDL-C, high density lipoprotein cholesterol; LDL-C, low density lipoprotein cholesterol; CK-MB, creatine kinase MB; cTnI, cardiac troponin I; cTnT, cardiac troponin T; LVEF, left ventricular ejection fraction.

**Table 2:** The comparison of AUC among PAPP-A, NT-IGFBP-4, CT-IGFBP-4 and other factors in predicting MACCE in AMI patients.

Patients with AMI (*n* = 341)						
	AUC	*p* Value	Cutoff Value	Sensitivity (%)	Specificity (%)	Youden Index (J)
PAPP-A, (ng/mL)	0.637 (0.534–0.740)	0.018	19.0	37.0	85.7	0.23
NT-IGFBP-4, (pg/mL)	0.633 (0.531–0.736)	0.022	735.3	74.1	55.7	0.33
CT-IGFBP-4, (pg/mL)	0.522 (0.404–0.640)	0.703	-	-	-	-
LVEF, (%)	0.633 (0.529–0.737)	0.022	0.41	70.4	53.2	0.24
Age (years)	0.678 (0.590–0.766)	0.002	56	81.5	49.0	0.31
eGFR (mL/min/1.73 m^2^)	0.719 (0.610–0.828)	<0.001	111	74.1	68.0	0.42
PAPP-A + LVEF ^a^ + Age + eGFR	0.742 (0.641–0.841)	<0.001	-	40.7	96.2	0.37
NT-IGFBP-4 + LVEF ^a^ + Age + eGFR	0.738 (0.641–0.841)	<0.001	-	74.1	60.5	0.35

^a^ means plus LVEF only in the patients with AMI. *p* means the *p* value of different factors when predicting one-year MACCE in different cohorts. Abbreviations: see Table 1, Figure 1.

**Table 3:** The comparison of AUC among different factors in predicting MACCE in patients with AMI and CKD.

Patients with AMI and CKD (*n* = 232)						
	AUC	*p* Value	Cutoff Value	Sensitivity (%)	Specificity (%)	Youden Index (J)
PAPP-A, (ng/mL)	0.667 (0.558–0.777)	0.009	11.7	65.2	61.2	0.27
NT-IGFBP-4, (pg/mL)	0.638 (0.517–0.759)	0.030	830.1	95.7	51.0	0.46
CT-IGFBP-4, (pg/mL)	0.537 (0.405–0.669)	0.559	-	-	-	-
LVEF (%)	0.615 (0.498–0.731)	0.072	-	-	-	-
Age (year)	0.641 (0.543–0.738)	0.027	58	95.6	32.1	0.28
eGFR (mL/min/1.73 m^2^)	0.721 (0.607–0.834)	0.001	103	87.0	52.7	0.39
PAPP-A + LVEF ^a^ + Age + eGFR	0.729 (0.618–0.840)	<0.001	-	47.8	93.4	0.42
NT-IGFBP-4 + LVEF ^a^ + Age + eGFR	0.726 (0.616–0.832)	<0.001	-	56.5	79.0	0.35

^a^ means plus LVEF only in patients with AMI and CKD. *p* means the *p* value of different factors when predicting one-year MACCE in different cohorts. Abbreviations: see Table 1, Figure 1.

**Table 4:** Univariable and multivariable Cox regression analyses for the composite MACCE and cardiovascular death.

	Patients with AMI (*n* = 341)					Patients with AMI and CKD (*n* = 232)			
	MACCE		Cardiovascular Death			MACCE		Cardiovascular Death	
	HR (95% CI)	*p*	HR (95% CI)	*p*		HR (95% CI)	*p*	HR (95% CI)	*p*
PAPP-A > 19.0 ng/mL					PAPP-A > 11.7 ng/mL				
Univariable	3.25 (1.49–7.09)	0.003	3.96 (1.59–9.86)	0.003	Univariable	2.81 (1.19–6.62)	0.018	2.97 (1.11–7.91)	0.030
Adjusted ^a^	2.48 (1.09–5.63)	0.030	2.87 (1.11–7.45)	0.030	Adjusted ^b^	1.85 (0.77–4.44)	0.169	1.80 (0.58–5.58)	0.308
NT-IGFBP-4 > 735.3 pg/mL					NT-IGFBP-4 > 830.1 pg/mL				
Univariable	3.37 (1.43–7.98)	0.006	3.28 (1.18–9.11)	0.023	Univariable	3.19 (1.40–7.28)	0.006	3.02 (1.19–7.66)	0.020
Adjusted ^a^	2.48 (1.03–6.02)	0.044	2.31 (0.81–6.62)	0.118	Adjusted ^b^	2.72 (1.16–6.41)	0.021	2.63 (1.01–6.84)	0.048

Model ^a^, adjusted for age, gender, diagnosis of STEMI, previous stroke, eGFR, LVEF in the AMI cohort. Model ^b^, adjusted for age, gender, diagnosis of STEMI, previous stroke and eGFR in the AMI and CKD cohort. Abbreviations: see Table 1, Figure 1.

## Data Availability

The data presented in this study are available on reasonable request from the corresponding author due to privacy and ethical restrictions. The datasets generated and/or analyzed during the current study are not publicly available because they contain information that could compromise the privacy of study participants.

## Supplementary Materials

The following supporting information can be downloaded at: https://www.mdpi.com/article/10.3390/jcm15155760/s1, Table S1: Bootstrap-Validated Performance of PAPP-A and NT-IGFBP-4 for MACCE; Table S2: Proportional-hazards assumption test results (Schoenfeld residual tests, MACCE).

## Abbreviations

The following abbreviations are used in this manuscript:

MACCE	major adverse cardiovascular and cerebrovascular events
BMI	body mass index
SBP	systolic blood pressure
DBP	diastolic blood pressure
HR	heart rate
CAD	coronary artery disease
AMI	acute myocardial infarction
STEMI	ST-segment elevation myocardial infarction
PCI	percutaneous coronary intervention
CABG	coronary artery bypass graft
ACEI	angiotensin-converting enzyme inhibitor
ARB	angiotensin-receptor blocker
FPG	fasting plasma glucose
HbA1c	hemoglobin A1c
eGFR	estimated glomerular filtration rate
hs-CRP	high-sensitivity C-reactive protein
TC	total cholesterol
TG	triglyceride
HDL-C	high-density lipoprotein cholesterol
LDL-C	low-density lipoprotein cholesterol
CK-MB	creatine kinase MB
cTnI	cardiac troponin I
cTnT	cardiac troponin T
LVEF	left ventricular ejection fraction
PAPP-A	pregnancy-associated plasma protein-A
NT-IGFBP-4	N-terminal fragments of human insulin-like growth factor binding protein-4
CT-IGFBP-4	C-terminal fragments of human insulin-like growth factor binding protein-4
