# Supplementary material for: Prognostic Values of Pregnancy-Associated Plasma Protein-A and IGF-Binding Protein-4 Fragments in Patients with Acute Myocardial Infarction
Source: J Clin Med. 2026 Jul 23;15(15):5760. doi: 10.3390/jcm15155760 (PMC13467400; doi:10.3390/jcm15155760)
Supplement: Supplementary file 1 [file jcm-15-05760-s001.zip › jcm-4395061-supplementary.pdf]

Supplementary Table S1. Bootstrap-Validated Performance of PAPP-A and NT-IGFBP-4 for MACCE

Cohort	Biomarker	C-index(95% CI)	Brier Score(95% CI)	Bootstrap Iterations
AMI cohort(n=341)	PAPP-A	0.622 (0.552–0.692)	0.030 (0.023–0.037)	1000
AMI cohort(n=341)	NT-IGFBP-4	0.681 (0.619–0.743)	0.029 (0.019 - 0.039)	1000
AMI&CKD (n=232)	PAPP-A	0.110 (0.048–0.172)	0.023 (0.016 - 0.030)	1000
AMI&CKD (n=232)	NT-IGFBP-4	0.654 (0.572–0.736)	0.036 (0.029 - 0.043)	1000

Note: Internal validation was performed via 1000 bootstrap iterations to correct for overfitting and estimate 95% confidence intervals. The optimism-corrected C-index measures model discrimination (predictive accuracy), while the Brier score measures overall model performance (calibration and accuracy), with lower values indicating better performance. Abbreviations see Table 1

Supplementary Table S2. Proportional-hazards assumption test results (Schoenfeld residual tests，MACCE)

Model	Global p-value
Overall AMI cohort-PAPP-A model	0.842
Overall AMI cohort-NT-IGFBP-4 model	0.308
AMI & CKD subgroup PAPP-A model	0.855
AMI & CKD subgroup-NT-IGFBP-4 model	0.502
